# Beyond codeine – the evidence landscape of conventional, natural, and emerging antitussive therapies: a systematic review and meta-analysis

**DOI:** 10.3389/fphar.2026.1756578

**Published:** 2026-03-02

**Authors:** Monika Marko, Rafał Pawliczak

**Affiliations:** Department of Immunopathology, Faculty of Medicine, Division of Biomedical Science, Medical University of Lodz, Lodz, Poland

**Keywords:** antitussive, codeine alternatives, cough suppression, natural therapies, P2X3

## Abstract

**Background:**

For decades, cough treatment has relied on centrally acting agents like codeine despite inconsistent efficacy. Advances in cough neurobiology enabled targeted therapies. Natural remedies remain widely used, though evidence for their effectiveness and safety is limited. This study aimed to compare evidence on the efficacy and safety of conventional, natural, and novel antitussives.

**Methods:**

A systematic review and meta-analysis of randomized clinical trials assessed cough frequency, Visual Analogue Scale (VAS), Leicester Cough Questionnaire (LCQ) and adverse events versus placebo.

**Results:**

Subgroup meta-analysis showed no significant differences between P2X3 antagonists, indicating a consistent class effect. All subgroups reduced chronic cough frequency [standardized mean difference (SMD) = −0.50, 95% confidence interval (CI) (−0.67–0.34), P = 0.0001], suggesting that the observed effect is a class-related response rather than a compound-specific effect.

**Conclusion:**

Conventional and natural antitussives show inconsistent efficacy. P2X3 receptor antagonists appear most promising, marking a shift beyond codeine toward targeted chronic cough therapies.

**Clinical Trial Registration:**

The meta-analysis was performed according to the protocol described in PROSPERO, identifier CRD420251172660.

## 1 Introduction

Cough is a vital protective reflex whose primary function is to generate high-velocity airflow, thereby clearing the airways and maintaining respiratory function (Morice et al., 2020; Lee et al., 2021). When this response becomes excessive or uncontrolled, however, it may evolve into a chronic condition that markedly affects quality of life and increases the healthcare burden (Jakusova and Brozmanova, 2023; Weinberger and Hurvitz, 2020). Children are considered to have a chronic cough when symptoms persist for more than 4 weeks, whereas in adults the threshold is 8 weeks (Weinberger and Hurvitz, 2020). The leading causes of chronic cough include asthma, chronic obstructive pulmonary disease (COPD), pulmonary fibrosis, eosinophilic bronchitis, chronic bacterial bronchitis (Weinberger et al., 2025), as well as upper airway cough syndrome and gastroesophageal reflux disease (GERD).

Increasing attention has been given to cough hypersensitivity syndrome (CHS), which is now recognized as a significant clinical entity accounting for a substantial proportion of patients with chronic cough in whom no clear underlying cause can be identified (Khan et al., 2024; Chung et al., 2016). This condition is characterized by an exaggerated cough response to otherwise innocuous stimuli (thermal, mechanical, or chemical), resulting from enhanced excitability and plasticity of airway sensory nerves, particularly involving vagal afferent pathways (Khan et al., 2024). Importantly, evidence suggests that, in addition to maladaptive neural plasticity, other pathophysiological mechanisms underlying cough hypersensitivity have not been sufficiently characterized (Song and Morice, 2017).

Other contributing factors comprise the use of antihypertensive agents such as angiotensin-converting enzyme (ACE) inhibitors, smoking, habit cough, and systemic disorders (Morice et al., 2020; Jakusova and Brozmanova, 2023). A so-called habit cough, more frequently observed in children and adolescents, is considered a functional disorder characterized by repetitive coughing without an identifiable organic cause, often influenced by behavioral or psychogenic factors (Weinberger et al., 2023). Identification of the etiologies of chronic cough is essential for effective management. In adults, the most common etiologies include cough-variant asthma (CVA), upper airway cough syndrome (UACS), and eosinophilic bronchitis (EB). In contrast, the etiologies of chronic cough in children differ, with protracted bronchitis (PB) predominating and asthma or CVA representing frequent etiologies in school-aged children. However, data on pediatric etiologies remain limited due to the restricted feasibility of diagnostic investigations (Yu et al., 2019).

Cough is one of the most common symptoms encountered in clinical practice, and despite significant progress in understanding its mechanisms, treating it remains a challenge for clinicians (Jakusova and Brozmanova, 2023; Rouadi et al., 2025).

For decades, the management of cough has relied on centrally acting agents, including codeine (Smith et al., 2006), dextromethorphan (Meeves et al., 2023), butamirate (Faruqi et al., 2014; Charpin and Weibel, 1990), and levodropropizine (Lee et al., 2022; Mannini et al., 2017), despite their limited and inconsistent clinical efficacy. Recent advances in the neurobiology of cough have led to the development of mechanism-based therapies, such as P2X3 receptor antagonists, that directly target peripheral cough pathways (Rouadi et al., 2025). This paradigm shift from symptom suppression to pathway-specific modulation marks a pivotal moment in antitussive research.

Alongside these agents, a wide range of natural or herbal remedies continues to be promoted for cough relief, reflecting patient preference for “natural” options (Murgia et al., 2021). It is estimated that nearly four billion people worldwide still rely primarily on herbal medicines for their healthcare needs (Wardani et al., 2023).

Natural cough medicines, often perceived as safer, offer an alternative to conventional over the counter (OTC) cough medications. It should also be emphasized that the market for so-called “natural” cough medicines is rapidly expanding, offering a wide range of preparations with diverse compositions and claimed mechanisms of action (Murgia et al., 2021). Moreover, because medicinal plants contain a variety of bioactive molecules that can simultaneously exert expectorant and antitussive effects, the classification of herbal cough remedies is difficult (Pourova et al., 2023).

An important aspect is the difficult-to-assess chemical composition of plant extracts, which can vary significantly depending on cultivation, harvesting, storage, and processing conditions. As a result, products sold under the same name may differ in composition and pharmacological activity (Murgia et al., 2021). Due to growing interest in natural cough remedies and the lack of consistent, high-quality scientific evidence, a critical assessment of the current state of knowledge is necessary.

Our study aimed to go beyond codeine and examine the current scientific evidence on the effectiveness and safety of conventional, natural, and novel antitussive therapies for the treatment of chronic cough. Therefore, we conducted a systematic review and meta-analysis to assess and compare the effectiveness and safety of antitussives. By integrating data from randomized clinical trials (RCTs), we aimed to provide an updated and comprehensive evidence synthesis to aid clinical decision-making in cough management.

## 2 Methods

### 2.1 Search strategy

This systematic review and meta-analysis was conducted in line with the Preferred Reporting Items for Systematic Reviews and Meta-Analyses (PRISMA) guidelines (Page et al., 2021). The study protocol was described in PROSPERO (CRD420251172660).

A comprehensive search was carried out across PubMed, the Cochrane Central Register of Controlled Trials (CENTRAL), Embase, and ClinicalTrials.gov up to October 2025. In addition, gray literature sources–such as conference abstracts, official reports, preprints, datasets, white papers, and patents were explored via Google Scholar. The complete search strategy is provided in Table 1. To ensure completeness, updated searches were performed prior to the final synthesis.

**TABLE 1:** Search strategy.

Database	Search strategy^a^
PubMed	((antitussive agents) OR (antitussive drugs) OR (cough treatment) AND ((efficacy)) AND ((safety) OR (adverse events) OR (side effects)) Filters applied: Randomized controlled trial, Clinical trial
Cochrane Central Register of Controlled Trials (CENTER)	Antitussive agents OR antitussive drugs OR cough treatment AND efficacy AND safety Filters applied: title abstract keyword, in Trials
ClinicalTrials.gov	(antitussive agents OR antitussive drugs OR cough treatment) AND (efficacy) AND (safety OR adverse events OR side effects) Filters applied: “Expert search”
Embase	(“antitussive agents”/exp OR “antitussive agents” OR “antitussive drugs” OR “cough treatment” OR “efficacy”/exp OR “efficacy” OR “safety”/exp OR “safety” OR “adverse events”/exp OR “adverse events” OR “side effects”) AND ([controlled clinical trial]/lim OR [randomized controlled trial]/lim) AND “coughing”/dm AND (“clinical trial”/de OR “controlled clinical trial”/de OR “controlled study”/de OR “double blind procedure”/de OR “human”/de OR “intervention study”/de OR “open study”/de OR “randomized controlled trial”/de OR “retrospective study”/de) AND (“article”/it OR “clinical trial”/it)
Google Scholar	((antitussive agents) OR (antitussive drugs) OR (cough treatment) AND ((efficacy)) AND ((safety) OR (adverse events) OR (side effects))

^a^ The searches were re-run before the final analysis to identify further studies and possibly include them.

The research question and selection criteria were formulated using the Population, Intervention, Comparison, Outcome, and Study design (PICOS) structure. The inclusion criteria were population: patients with cough, intervention: antitussive drugs, comparison: placebo, outcomes: cough count, change in cough frequency, Visual Analogue Scale (VAS), Leicester Cough Questionnaire (LCQ) and adverse events (AEs), and study design: randomized clinical trials (RCTs), non-randomized clinical trials, real-life trials, observational trials, open-label trials, and prospective trials.

The following exclusion criteria were formulated: review article, systematic reviews, meta-analysis, case series, case report, articles with insufficient information and data, articles published in languages other than English, original articles where specific data and outcomes could not be extracted, original articles that do not include outcomes of interest and retracted articles.

### 2.2 Study selection and data extraction

The study selection process was conducted independently by two reviewers in parallel. Titles and abstracts identified through database searches were first screened for relevance, and duplicate records were excluded. Full texts of potentially eligible studies were then assessed in detail to determine whether they met the predefined inclusion criteria. To minimize selection bias, eligibility was judged independently by both reviewers, and any discrepancies were resolved through discussion until consensus was achieved.

Data extraction was performed independently using standardized forms. Extracted information included study identifiers (title, authors, institutional affiliation, and registration number), study design, duration, year of publication, intervention details, sample size, participant age, underlying condition, and reported outcomes. The two sets of extracted data were subsequently compared for accuracy and consistency.

All included studies were further assessed for missing or unclear data. Where required, efforts were made to clarify information or standardize reporting. In accordance with Cochrane guidelines (Higgins et al., 2024), results expressed as confidence intervals (CIs) or standard errors of the mean (SEM) were converted to means and standard deviation (SD) to ensure comparability across studies.

### 2.3 Assessment of the risk of bias and methodological quality

Risk of bias was assessed independently by two reviewers using the Cochrane Risk of Bias tool in Review Manager 5.4. Seven domains were evaluated (sequence generation, allocation concealment, blinding, incomplete data, selective reporting, and other bias), with studies rated as low, unclear, or high risk. Potential conflicts of interest were also considered in relation to funding sources. Disagreements were resolved by consensus, and an attrition rate above 10% was regarded as potentially influencing study validity.

### 2.4 Statistical methods

All extracted data were analyzed using Review Manager (RevMan) version 5.4. Continuous outcomes were expressed as standardized mean difference (SMD) with 95% confidence intervals (CI), and dichotomous outcomes as risk ratios (RR) with CI. Given the expected heterogeneity, pooled analyses were performed using the Mantel–Haenszel random-effects model. Heterogeneity was assessed with Cochran’s Q test and the I^2^ statistic, with P < 0.05 considered significant. Results were presented as forest plots, and SMDs were interpreted according to Cohen’s thresholds (0.2 small, 0.5 moderate, ≥0.8 large).

### 2.5 Additional analyses

The certainty of evidence for the outcomes included in the quantitative synthesis was appraised using the Grading of Recommendations Assessment, Development, and Evaluation (GRADE) approach (Schünemann et al., 2023; Guyatt et al., 2013). This assessment considered potential limitations related to risk of bias, inconsistency, indirectness, imprecision, and publication bias. Evidence quality was classified as high, moderate, low, or very low, following the standard GRADE framework, and findings were summarized in a Summary of Findings (SoF) table (Guyatt et al., 2013). The potential for publication bias was further examined through visual inspection of funnel plots.

## 3 Results

### 3.1 Included studies

**FIGURE 1:**
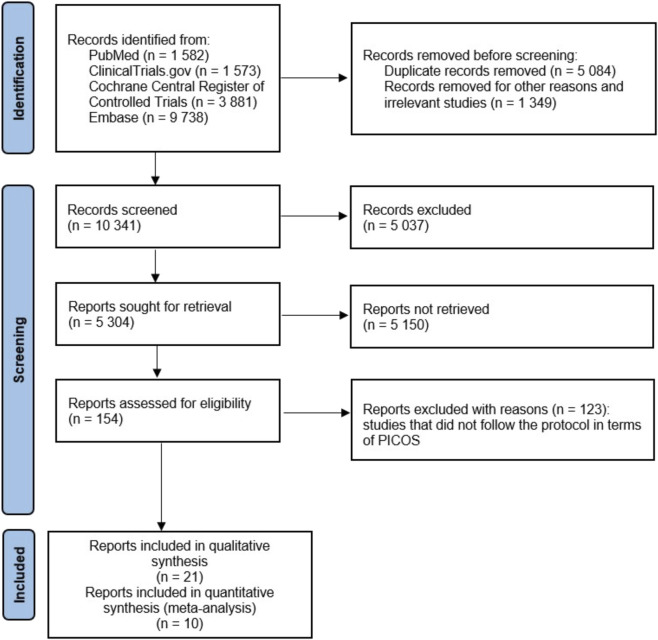
Flowchart of the screening procedure. PICOS: Population, Intervention, Comparison, Outcome and Study design.

Figure 1 (Page et al., 2021) illustrates the study selection process. Table 2 summarizes the characteristics of the studies included in the meta-analysis (Dicpinigaitis et al., 2023; Abdulqawi et al., 2015; Smith et al., 2020a; Smith et al., 2020b; Smith et al., 2025; Birring et al., 2024; McGarvey et al., 2023; Niimi et al., 2022; Morice et al., 2021) and a systematic review (Smith et al., 2006; Meeves et al., 2023; Mannini et al., 2017; Canciani et al., 2014; Carnevali et al., 2021; Ghaemi et al., 2020; Pan et al., 2025; Paul et al., 2014; Qasemzadeh et al., 2015; Kemmerich et al., 2006; Kemmerich, 2007; Núñez et al., 2024; Watson et al., 2008; Willcox et al., 2021; Lee et al., 2000; Eccles et al., 1992; Freestone et al., 1996; Bhattacharya et al., 2013; Bruschi et al., 2003; McGarvey et al., 2022; Barth et al., 2015). Following study selection, quantitative synthesis was feasible exclusively for chronic cough, as no eligible studies investigating acute cough met the predefined inclusion criteria.

**TABLE 2:** Characteristics of studies included in a systematic review and meta-analysis.

Studies included in a meta-analysis				
Study (author, year)/Design	Duration	Intervention	Participants N, age, condition	Main outcomes and measures
(Dicpinigaitis et al., 2023) PAGANINI: a randomized, double-blind, parallel-group, placebo-con- trolled, dose-finding	12 weeks	Twice daily: Eliapixant 25 mg 75 mg 150 mg placebo	N = 310, ≥18 refractory chronic cough (RCC)	PO: Change from baseline in 24-h cough count after 12 weeks of intervention SO: ≥30% reduction in 24-h cough count at 12 weeks Change in 24-h cough count at 2, 4, 8 weeks PO: Change in awake coughs/hour at 2, 4, 8, 12 weeks Change in cough severity (VAS) at 12 weeks; ≥30-unit VAS reduction Change in cough-related QoL (LCQ) at 12 weeks; ≥1.3-point LCQ increase Treatment-emergent AEs
(Abdulqawi et al., 2015) a randomized, double-blind, placebo-controlled phase 2 study	8 weeks (two 2-week treatment periods + 2-week washout + 2-week follow-up)	Twice daily: AF-219 600 mg placebo	N = 24 Mean age: 54.5 years (24–70) refractory chronic cough (RCC)	PO: Daytime cough frequency, night-time and 24-h cough frequency assessed SO: Changes in cough severity VAS (day/night), urge-to-cough VAS, and CQLQ scores; global rating of change (15-point scale) for cough severity and frequency Treatment-emergent AEs
(Smith et al., 2020a) I and II Two randomized, double-blind, placebo-controlled, two-period crossover, dose-escalation studies	∼5–8 weeks (two 16-day treatment periods + washout)	Gefapixant BID 7.5–200 mg; 4-day escalation placebo	N = 59 mean age 63 years (47–76) refractory chronic cough (RCC)	PO: Awake cough freq. (VitaloJAK) SO: VAS, cough diary, LCQ AEs; physical examination; vital signs; ECG; blood and urine analyses
(Smith et al., 2020b) III a randomized, double-blind, controlled, parallel-group, phase 2b trial	12-week	Gefapixant BID 7.5–50 mg placebo	N = 253 Age range: 18-80 refractory chronic cough (RCC) or unexplained chronic cough	PO: Change from baseline in awake objective cough frequency after 12 weeks (day 84) SO: Change in awake/24-h/sleep cough freq. (4–12 w, FU 14 weeks); VAS; LCQ; responder rates (≥30/50/70%); PGIC, CGIC. Vital signs, laboratory assessments, and AEs
(Smith et al., 2025) SOOTHE: a phase 2b, randomized, placebo-controlled trial	4 weeks	camlipixant 12.5, 50, or 200 mg twice daily or placebo for 4 weeks	N = 310 Age range: 18-80 refractory chronic cough (RCC)	PO: Change in 24-h cough frequency (Day 28, VitaloJAK) SO: Change in 24-h (Day 15), awake/night frequency (Days 15, 28); responders (>30/50/70%); CS-VAS; LCQ (Days 15, 29) AEs, TEAEs, abnormal exam/ECG/vital signs
(Smith et al., 2020b) A randomized placebo- controlled study	∼3 weeks total (two 1-day periods, 48-h washout, 2-weeks follow-up)	single dose gefapixant 100 mg or placebo	N = 24 Age range: 18–80 chronic cough	PO: Cough challenge thresholds C2 (≥2 coughs) and C5 (≥5 coughs) for each challenge agent, averaged across 1-, 3-, and 5-h post-exposure SO: VAS (cough severity, urge-to-cough), 24-h cough freq. (ambulatory), HARQ total score (0–70) Serious AEs, physical examinations, vital signs, 12-lead ECGs, and clinical laboratory tests
(Birring et al., 2024) a phase 3b, randomized, multicentre, double-blind, placebo-controlled trial	≈16 weeks total (2-week run-in +12-week treatment + 2-week follow-up)	Gefapixant 45 mg twice daily or placebo	N = 375 Mean age: 56.4 years (SD 11.4) chronic cough and cough-induced stress urinary incontinence	PO: Percentage change from baseline in 7-day average of daily CSUI episodes at week 12 (recorded in incontinence diary) SO: Change from baseline in CSD, total daily incontinence episodes, cough severity VAS, I-QOL, work productivity and activity impairment, and percentage of participants improved on the PGIC scale AEs
(McGarvey et al., 2023) phase 3b, double-blind, placebo-controlled, parallel group, multicenter study	≈16 weeks total (2-week screening +12-week treatment + 2-week follow-up)	gefapixant 45 mg BID or placebo	N = 415 ≥18 years of age mean age 52.5 years recent-onset refractory chronic cough (RCC) or unexplained chronic cough (UCC)	PO: change from baseline at week 12 in LCQ total score SO: The change from baseline in Cough Severity VAS score at week 12 and the CSD AEs
(Niimi et al., 2022) a phase 2a, randomized, double-blind, placebo-controlled, crossover, multicentre study	Approximately 6–7 weeks (2 weeks of treatment, 2–3-week washout period, followed by another 2 weeks of treatment in the crossover phase)	oral sivopixant 150 mg or placebo once daily for 2 weeks	N = 31 Age range: 20–75 years refractory chronic cough (RCC)	PO: Ratio of daytime coughs per hour after 2 weeks vs. baseline SO: Ratios of coughs per hour (24 h, nighttime, awake, asleep) vs. baseline Change in J-LCQ and % achieving MID (≥1.3 points) Change in cough severity (VAS) Change in EQ-5D-5L and EQ-VAS AEs and blood pressure, pulse rate, ECG and clinical laboratory tests
(Morice et al., 2021) a randomized, placebo-controlled, crossover phase 2a study	∼9–10 weeks (two 3-week periods + 3–4 weeks washout)	Period A: placebo 2 weeks: eliapixant 10 mg BID 1 week; Period B: eliapixant 50/200/750 mg BID (1 week each)	N = 40 >18 years refractory chronic cough (RCC)	PO: Change in cough frequency per hour (24-h objective monitoring, VitaloJAK) at baseline (day 1) and end of each treatment week (days 7, 14, 21); awake and asleep cough frequencies also assessed SO: Patient-reported cough severity (VAS, 100 mm) and cough-related quality of life (LCQ) Frequency and severity of AEs
Studies included in a systematic review				
Study ID	Duration	Intervention	Participants N, age, condition	Main outcomes and measures
(Lee et al., 2000) a double-blind, stratifed, rando- mized and parallel group design study	Single-day sessions (3 h follow-up)	Dextromethorphan 30 mg Placebo	N = 44 Age range: 18-60 cough associated with acute upper respiratory tract infection	Not specifically reported
(Eccles et al., 1992) a randomized, stratified, double- blind, parallel group, placebo-controlled study	3-h laboratory assessment + 4-day home phase	Codeine 30 mg/10 mL q.d.s. (total 120 mg/day) placebo syrup	N = 91 Mean age: 23 years, (18-71) cough associated with acute upper respiratory tract infection	Not specifically reported
(Smith et al., 2006) double-blind, randomized, placebo- controlled study	2 single-day periods, 1 week apart	Codeine phosphate 60 mg or matched placebo	N = 21 Mean age: 67.7 years cough in chronic obstructive pulmonary disease	PO: Time spent coughing (cough seconds, cs) during 10-h daytime and overnight recordings (objective cough frequency) SO: Not specifically reported
(Freestone et al., 1996) a double-blind, stratified, placebo-controlled, parallel-group, clinical trial	2 days	50 mg codeine or matched placebo in capsule form	N = 83 Mean age: 23.5 years cough associated with common cold or acute URTI	cough sound-pressure levels (CSPLs) measured on a sound meter; subjective scores of coughs seventy; and cough frequency recorded by means of a microphone connected to an ink-pen recorder
(Bhattacharya et al., 2013) randomized double-blinded placebo-controlled trial	dextromethorphan 5 mg promethazine 0.5 mg/kg placebo	3 days	N = 120 median age: 5.5 years nocturnal cough during URTI	Symptom scores AEs
(Meeves et al., 2023) multiple‐dose, double‐blind, placebo‐controlled, randomized, pilot clinical study	4 days	Dextromethorphan 15 mg/10 mL or placebo	N = 131 Age range: 6–11 years acute cough due to the common cold	PO: Total cough count during the first 24 h of treatment SO: Cough rate (counts/h) during daytime, nighttime, and individual dosing intervals Total time with cough events over 24 h Change from baseline for cough severity, frequency, and impact on sleep (5-point scales) Change in Child Global Question Subject/parent satisfaction with treatment AEs
(Bruschi et al., 2003) a double-blind, randomized, cross-over trial	Approximately 7–13 days (two treatment sessions of 10 consecutive doses each, separated by a 3–7-day washout period)	Levodropropizine 60 mg (20 drops) or matching placebo, administered orally three times daily (t.i.d.) for 10 consecutive doses	N = 26 Age range: 18–45 years healthy volunteers and patients with chronic respiratory impairment associated with chronic obstructive pulmonary disease	Bronchodilator reversibility by FEV_1_ change after salbutamol 200 μg Spirometry and arterial blood gases measured at baseline and post-inhalation Respiratory parameters recorded at rest Ventilatory response to hypercapnia assessed with 0%–7% CO_2_ AEs and general tolerability evaluation by physical examination, vital signs (blood pressure, heart rate), ECG
(Mannini et al., 2017) Randomized clinical trial	Approximately 6–9 days (three treatment sessions separated by 48–72-h washout periods)	60 mg Levodropropizine, or 15 mg Dihydrocodeine or matching placebo	N = 24 Age range: 39–70 years Chronic cough	Ventilatory response to CO_2_ rebreathing
(McGarvey et al., 2022) two double-blind randomized, parallel-group, placebo-controlled, phase 3 trials (COUGH-1 and COUGH-2)	12 weeks (COUGH-1) and 24 weeks (COUGH-2)	gefapixant 15 mg twice per day, or gefapixant 45 mg twice per day or placebo	COUGH-1 N = 732 COUGH-2 N = 1,314 Mean age 59.0 years (12.6) in COUGH-1 Mean age 58.1 years (12.1) in COUGH-2 refractory chronic cough or unexplained chronic cough	PO: Change from baseline in 24-h cough frequency (weeks 12/24) Secondary: Change in awake cough frequency ≥30% reduction in 24-h cough frequency ≥1.3-point LCQ improvement Reductions in Cough Severity Diary and VAS scores Exploratory: 50% and 70% cough-frequency responders AEs, vital signs, physical examination, and laboratory tests
(Barth et al., 2015) a comparative, randomized, double-blind, placebo-controlled study	5 days	*Justicia adhatoda, Echinacea purpurea* and *Eleutherococcus senticosus extracts* -KJ (30 mL/day Bromhexine 1.6 mg/mL and Placebo	N = 177 KJ Mean age ±SD: 34.8 ± 11.39 Bromhexine Mean age ±SD: 32.1 ± 10.38 Placebo Mean age ±SD: 31.9 ± 11.57 patients with acute upper respiratory tract infection	PO: efficacy of treatment (Cough Relief Index), cough frequency SO: Hematological and immunological parameters AEs
(Canciani et al., 2014) a randomized, multicenter, double blind, placebo-controlled clinical trial	8 days	Grintuss 4 doses a day, 5 mL each, and placebo	N = 102 Age range: 3–5 years cough persisting more than 7 days	PO: The changes in the day- and night-time cough score SO: Not specifically reported AEs
(Carnevali et al., 2021) a randomized, double blind placebo-controlled clinical trial	8 days	KalobaTUSS 4 doses daily in 5 mL per dose and placebo	N = 106 Mean age: (months) 53.0 ± 12.0 acute cough	PO: The change in the night-time cough score before treatment and nocturnal scores obtained after the first, fourth and eighth nights of treatment, and the change in the diurnal time cough score before treatment and scores obtained after the first, fourth and eighth days of treatment SO: The secondary outcome was the safety of the syrup when administered to children with acute cough for 8 days, which was evaluated by recording potential adverse reactions to treatment AEs
(Ghaemi et al., 2020) A randomized, double-blinded, placebo- controlled clinical trial	15 months	Licorice pastilles one pastille of 300 mg (licorice, acacia, and tragacanth) or a placebo three times daily for 2 weeks	N = 70 Age range:18-70 chronic cough	PO: a decrease in the daily cough scores during the 2-week treatment period SO: the assessment of the quality-of-life measure of chronic cough ac- cording to the LCQ scores AEs
(Pan et al., 2025) A multicenter, randomized, double-blinded, placebo-controlled trial	28-day follow-up 14-day treatment	Suhuang Zhike capsules 1.35 g of SHZK granules or placebo	N = 132 Age range: 18-60 Asthma	PO: Change in ACT scores on day 42 relative to baseline, reflecting asthma control status SO: Changes in cough symptom scores on days 7, 14, and 42 relatives to the baseline. Changes in VAS scores of coughs, wheezing, chest tightness, and shortness of breath on days 7, 14, and 42 relatives to the baseline AEs
(Paul et al., 2014) Randomized clinical trial	13 months	Agave nectar 3 mL (age 2–5 months); 4 mL (age 6–23 months); 5 mL (age 24–47 months) or placebo	N = 120 The mean age: 22.9 (14.0) months nonspecific acute cough	The outcome of between-night change Cough frequency, cough severity, cough bothersomeness, congestion severity, rhinorrhea severity, and cough effect on child and parent sleep AEs
(Qasemzadeh et al., 2015) double-blind, randomized controlled clinical trial	7 months	*Viola odorata* Flower Syrup And placebo administered for 5 days; 2.5 cc three times daily for children aged 2–5 years, and 5 cc three times daily for children ≥5 years. Each 100 cc of violet syrup contained 12 g of dried *Viola* flower	N = 182 Age range: 2–12 years old Asthma	Evaluation in terms of the duration until cough suppression was achieved AEs
(Kemmerich et al., 2006) a double-blind, placebo- controlled, multicentre Phase IV study	5 months	Thyme-ivy syrup 5.4 mL three times daily for 11 days (15 g thyme +1.5 g ivy extract/100 g); placebo identical in appearance and flavour	N = 363 Thyme-ivy combination Mean age ±SD: 43.4 ± 17.7 Placebo Mean age ±SD: 41.5 ± 17.3 acute bronchitis with productive cough	PO: Change in mean frequency of daytime coughing fits (Days 7–9 vs. Day 1) SO: Reduction in coughing fits (AUC), time to 50% reduction, proportion without coughing fits on Day 9, relative reduction at Day 9, investigator-assessed response, change in BSS, mucus expectoration, sleep disturbance, and general wellbeing (all assessed by diary or rating scales) AEs, vital signs, patient’s and investigator’s global judgement of tolerability at study end
(Kemmerich, 2007) a double-blind, placebo- controlled, multicentre Phase IV study	5 months	Thyme–primrose tablets 160 mg thyme +60 mg primrose extract/tablet or placebo	N = 362 Thyme-primrose combination Mean age ±SD: 41.9 ± 14.9 Placebo Mean age ±SD: 43.5 ± 16.4 acute bronchitis with productive cough	PO: Change in mean frequency of daytime coughing fits (Days 7–9 vs. Day 1) SO: Reduction in coughing fits (AUC), time to 50% reduction, cough-free rate (Day 9), relative reduction at Day 9, investigator-assessed response, BSS change, mucus expectoration, sleep disturbance, and general wellbeing AEs, vital signs
(Núñez et al., 2024) A pilot, randomized, double-blind, placebo- controlled, multicenter clinical trial	14-day study: 7-day treatment, and 7-day safety follow-up	Sediflu syrup 10 mL BID for 7 days	N = 56 Mean age ±SD: 8.65 ± a 2.14 dry and/or productive cough	PO: Change in day- and night-time CCS. SO: Tolerability (Paul’s Night Cough Questionnaire) AEs
(Watson et al., 2008) randomized, placebo-controlled, double-blind trial	4 weeks	purple passion fruit (PFP) peel extract (150 mg/d) or placebo pills	N = 43 PFP Mean age ±SD: 36.4 ± 15.7 Placebo Mean age ±SD: 35.8 ± 11.5 Asthma	Asthma symptoms and spirometry (FVC, FEV_1_) at baseline and weekly AEs
(Willcox et al., 2021) Feasibility double-blind randomized placebo-controlled clinical trial	9 months	Pelargonium sidoides extract (EPs®7630, Kaloba®) Oral solution: 10 g (≈9.75 mL) contains 8.0 g extract; Film-coated tablet: 20 mg dry extract (1:8–10)	N = 132 average age: 56–59 years acute cough due to lower respiratory tract infection	Feasibility (recruitment, diary return/completion, antibiotic use, symptom duration/resolution, medication compliance), clinical (symptom duration, resolution at Day 7), and health-economic (EQ-5D-5L, NHS resource use, costs) AEs

CSUI: cough associated stress urinary incontinence, ECG: electrocardiogram, EQ-5D-5L: EuroQol 5 Dimensions, 5 Levels, FEV_1_: Forced Expiratory Volume in 1 s, FU: follow-up, FVC: forced vital capacity, HARQ: hull airway reflux questionnaire, J-LCQ: japanese version of the leicester cough questionnaire, LCQ: leicester cough questionnaire, MID: minimal important difference, NHS: national health service, PGIC: patient global impression of change, PO” primary endpoint, PROs: Patient-Reported Outcomes, PFP: purple passion fruit, q.d.s.: four times daily, SO: secondary endpoint, t.i.d.: three times daily, UCC: unexplained chronic cough, VAS: visual analogue scale.

No additional records were identified through the gray literature search (Google Scholar), and no duplicates were detected. The updated search yielded no further eligible studies for inclusion in the systematic review or meta-analysis.

**FIGURE 2:**
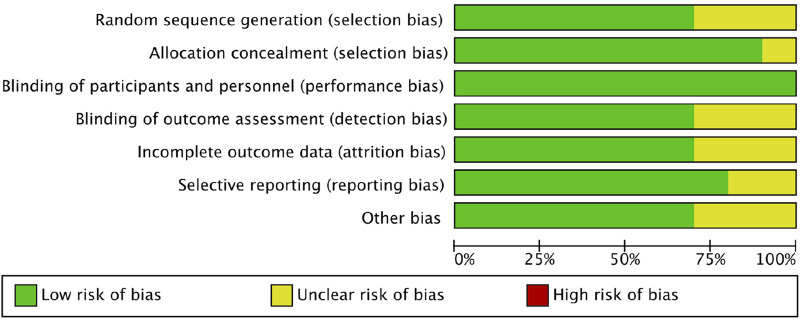
Risk of bias summary. Review authors’ judgements about each risk of bias item for each included study.

**FIGURE 3:**
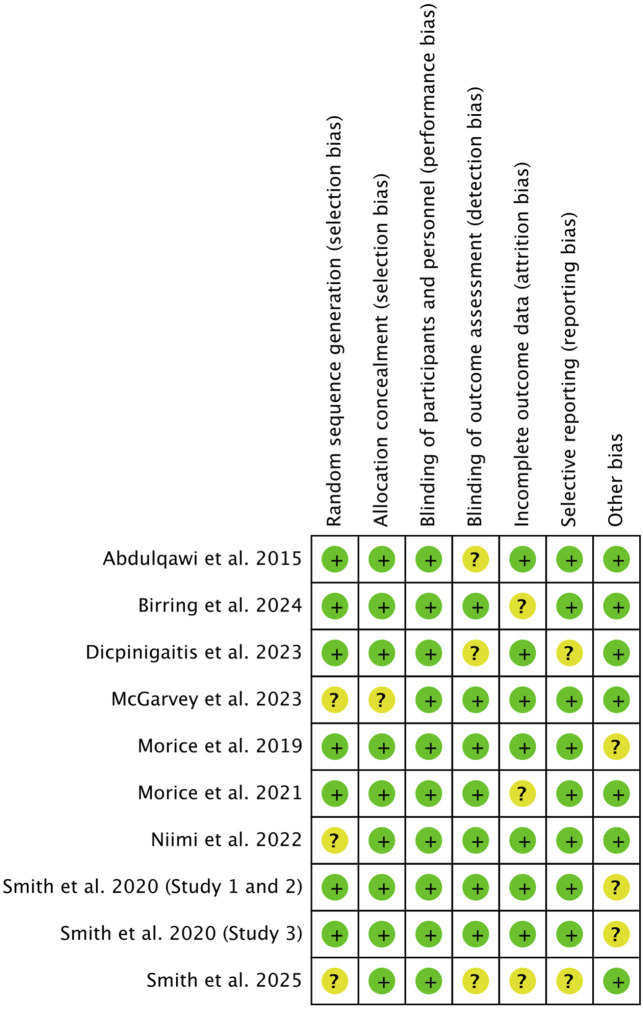
Risk of bias graph. Review authors’ judgements about each risk of bias item presented as percentages across studies.

The pooled risk of bias assessment (Figures 2, 3) confirmed that most studies were of low risk. Qualitative evaluation did not suggest substantial publication bias. Given the limited number of studies (n < 10), funnel plots were used for illustrative purposes only, as recommended by Cochrane guidelines (Higgins et al., 2024). Visual inspection of the plots (Supplementary Figures S1–S4) showed no marked asymmetry, though the small sample precludes definitive conclusions.

### 3.2 P2X3: cough count (24 h frequency, chronic cough)

**FIGURE 4:**
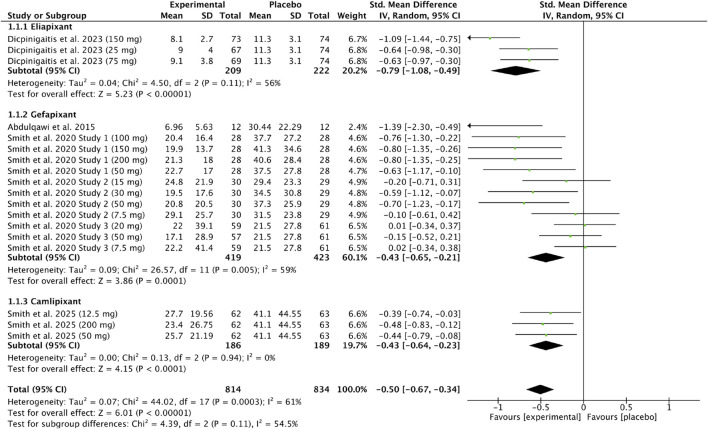
Standardized mean difference in 24-h cough frequency following P2X3 antagonist treatment in patients with chronic cough compared with placebo.

Five studies (Dicpinigaitis et al., 2023; Abdulqawi et al., 2015; Smith et al., 2020a; Smith et al., 2020b; Smith et al., 2025) were included in the subgroup meta-analysis of 24-h cough frequency after P2X3 antagonist administration versus placebo. Due to varying dosages and study stages, data were analyzed in three subgroups: eliapixant, gefapixant, and camlipixant. The pooled analysis (814 experimental vs. 834 placebo participants) showed a significant reduction in cough frequency [SMD = −0.50, 95% CI (–0.67, −0.34), P < 0.00001; I^2^ = 61%]. No significant subgroup differences were observed (P = 0.11; I^2^ = 54.5%), with eliapixant demonstrating the greatest effect [SMD = −0.79, 95% CI (–1.08, −0.49), P < 0.00001; I^2^ = 56%]. Results are presented in Figure 4.

### 3.3 Gefapixant: visual analogue scale (chronic cough)

**FIGURE 5:**
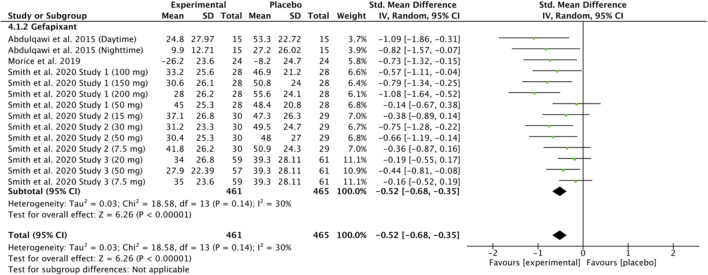
Standardized mean difference in Visual Analogue Scale (VAS) scores following gefapixant treatment in patients with chronic cough compared with placebo.

Four studies (Dicpinigaitis et al., 2023; Abdulqawi et al., 2015; Smith et al., 2020a; Smith et al., 2020b) were included in the meta-analysis of Visual Analogue Scale (VAS) scores after gefapixant versus placebo. Data from 461 experimental and 465 placebo participants were analyzed, with some studies divided by dosage or observation period. The pooled analysis showed a significantly lower VAS score in the gefapixant group [SMD = −0.52, 95% CI (–0.68, −0.35), P < 0.00001; I^2^ = 30%]. Results are presented in Figure 5.

### 3.4 Gefapixant: leicester cough questionnaire (chronic cough)

**FIGURE 6:**
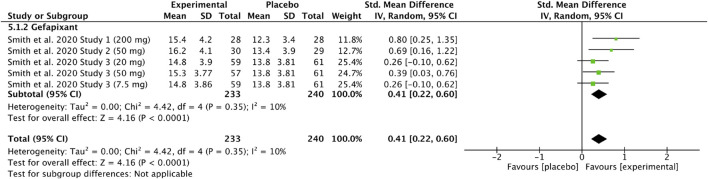
Standardized mean difference in Leicester Cough Questionnaire (LCQ) scores following gefapixant treatment in patients with chronic cough compared with placebo.

Two studies (Smith et al., 2020a; Smith et al., 2020b) were included in the meta-analysis of Leicester Cough Questionnaire (LCQ) scores after gefapixant versus placebo. Data from 233 experimental and 240 placebo participants were analyzed, with study parts divided by dosage or design. The pooled analysis showed a significantly higher LCQ score in the gefapixant group [SMD = 0.41, 95% CI (0.22, 0.60), P < 0.0001; I^2^ = 10%]. Results are shown in Figure 6.

### 3.5 P2X3: adverse events (chronic cough)

**FIGURE 7:**
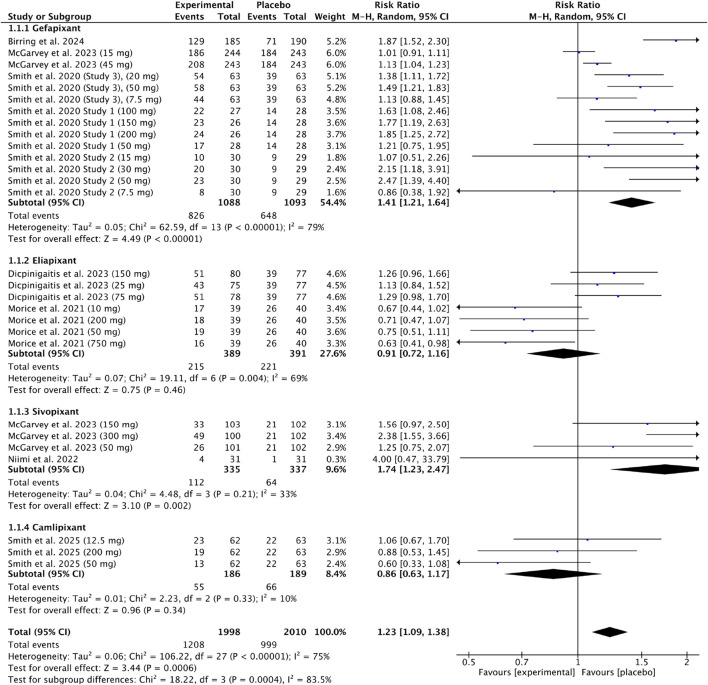
Risk ratio of adverse events (AEs) following P2X3 antagonist treatment in patients with chronic cough compared with placebo.

Eight studies (Dicpinigaitis et al., 2023; Smith et al., 2020a; Smith et al., 2020b; Smith et al., 2025; Birring et al., 2024; McGarvey et al., 2023; Niimi et al., 2022; Morice et al., 2021) were included in the meta-analysis of adverse events (AEs) after P2X3 antagonist administration versus placebo. Data from 1998 experimental and 2010 placebo participants were analyzed across four subgroups (gefapixant, eliapixant, sivopixant, camlipixant). The pooled analysis showed a higher incidence of AEs in the experimental group [RR = 1.23, 95% CI (1.09, 1.38), P = 0.0006; I^2^ = 75%], with significant subgroup differences (P = 0.00004; I^2^ = 83.5%), indicating that AEs frequency varied by the type of P2X3. Results are presented in Figure 7.

### 3.6 Results of additional analyses

The certainty of evidence for all analyzed outcomes was evaluated according to the GRADE methodology (Guyatt et al., 2013). The summary of findings and corresponding GRADE ratings are presented in Table 3.

**TABLE 3:** Summary of findings (SoF) of meta-analysis using working group grades of evidence (GRADE).

Continuous data					
Patients, interventions, comparators	Number of participants/studies		Intervention vs. comparator standardized mean difference (95% CI)	Comparator	Certainty of the evidence (GRADE)
Eliapixant vs. placebo	209 vs. 222/1 study (divided into three)		−0.79 [−1.08, −0.49] Lower cough count in the experimental group. a moderate-to-large effect size, likely reflecting a meaningful clinical improvement^a^	The mean cough count (24 h frequency)	High ⊕⊕⊕⊕
Gefapixant vs. placebo	419 vs. 423/4 studies (3 studies divided into several parts)		−0.43 [−0.65, −0.21] Lower cough count in the experimental group A small-to-moderate effect size, suggesting a modest but potentially meaningful clinical improvement^a^	The mean cough count (24 h frequency)	Moderate ⊕⊕⊕○
Camlipixant vs. placebo	186 vs. 189/1 study (divided into three)		−0.43 [−0.64, −0.23] Lower cough count in the experimental group A small-to-moderate effect size, indicating a modest but potentially meaningful clinical improvement^a^	The mean cough count (24 h frequency)	Moderate ⊕⊕⊕○
Gefapixant vs. placebo	461 vs. 465/4 studies (3 of them divided into several parts)		−0.52 [−0.68, −0.35] Lower VAS in the experimental group A moderate effect size, suggesting a clear and clinically relevant improvement^a^	The mean VAS	High ⊕⊕⊕⊕
Gefapixant vs. placebo	233 vs. 240/3 studies (1 of them divided into several parts)		0.41 [0.22, 0.60] Higher LCQ in the experimental group A small-to-moderate effect size, suggesting a modest but noticeable clinical difference^a^	The mean LCQ	High ⊕⊕⊕⊕
Dichotomous data					
Outcomes	Absolute Effect (95% CI)		Relative effect (95% CI)	Number of studies	Certainty of the evidence (GRADE)
	Risk with placebo	Risk with experimental			
Occurrence of adverts events Gefapixant vs. placebo	648 per 1,000	914 (785-1,000) per 1,000	RR 1.41 [1.21, 1.64]	4 studies (3 of them divided into several parts)	Low ⊕⊕○○
	Absolute difference per 1,000: +266 (+137 do +352) The relative risk of 1.41 (1.21–1.64) indicates a significant 41% increase in risk				
Occurrence of adverts events Eliapixant vs. placebo	221 per 1,000	217 (190–245) per 1,000	RR 0.98 [0.86, 1.11]^b^	3 studies divided into several parts	Low ⊕⊕○○
	Absolute difference per 1,000: −4 (−31 to +24) The relative risk of 0.98 (0.86–1.11) suggests a small (2%) reduction in risk, but the result is not statistically significant				
Occurrence of adverts events Sivipixant vs. placebo	64 per 1,000	113 (86–147) per 1,000	RR 1.76 [1.35, 2.30]	2 studies (1 of them divided into several parts)	Moderate ⊕⊕⊕○
	Absolute difference per 1,000: +49 (+22 to +83) The relative risk of 1.76 (1.35–2.30) indicates a statistically significant 76% increase in risk				
Occurrence of adverts events Camlipixant vs. placebo	66 per 1,000	56 (42–75) per 1,000	RR 0.85 [0.63, 1.14]^b^	1 study divided into several parts	Low ⊕⊕○○
	Absolute difference per 1,000: −10 (−24 to +9) The relative risk of 0.85 (0.63–1.14) suggests a 15% relative reduction in risk But the result is not statistically significant				

95% CI: 95% Confidence interval; GRADE: working group grades of evidence, LCQ: leicester cough questionnaire, RR: risk ratio, VAS: visual analogue scale.

Explanations:

^a^ According to Cohen’s convention.

^b^ Statistically insignificant outcome.

## 4 Discussion

This systematic review and meta-analysis included centrally, and peripherally acting medications commonly used to treat cough. The analysis aimed to assess their efficacy and safety based on the results of available RCTs. It should be emphasized that this study did not present quantitative data for codeine, butamirate, dextromethorphan, and levodropropizine. Only qualitative analysis was possible for these antitussives (Table 2). The inability to conduct a meta-analysis of the data from the included studies confirms previous observations that the available studies are characterized by significant heterogeneity. Furthermore, their conclusions are inconsistent. Consequently, it is impossible to draw clear conclusions about the efficacy of these medications in treating cough.

As shown in Table 2, the observed heterogeneity results from significant differences across studies, including drug doses used, therapy duration, methods of efficacy assessment, and heterogeneity in the patient population (age, cough etiology, and comorbidities). These factors contributed to the discrepancy in results and limited the comparability of data in this meta-analysis.

Eccles et al. (1992) in a study using a 30 mg codeine syrup, no significant differences in cough frequency or subjective cough severity were observed between the codeine and placebo groups, either in the laboratory or during home monitoring. These results suggest that cough mechanisms during viral infections may be poorly responsive to opioid antitussives, underscoring the need to explore alternative agents with distinct mechanisms of action.

Moreover, a study (Smith et al., 2006) in patients with stable COPD demonstrated that codeine, although considered a standard antitussive, did not significantly affect cough frequency or subjective cough severity compared with placebo. Despite a slight decrease in cough time after codeine administration compared to baseline, this difference was not statistically significant compared to the control group. These results suggest that cough mechanisms in COPD may be resistant to the effects of opioid antitussives, questioning the validity of using codeine as a reference point in studies of new antitussive agents.

Similarly, a clinical trial in children (Bhattacharya et al., 2013) with upper respiratory tract infection (URTI) found that neither dextromethorphan nor promethazine was more effective than placebo at reducing nocturnal cough or improving sleep quality. Analogously, a study of adults with URTI (Lee et al., 2000) showed no significant advantage over placebo in relieving cough.

These results emphasize that the routine use of antitussives in children and adults with acute upper respiratory tract infection is unjustified, and the effectiveness of both opioid and non-opioid antitussives remains limited. The antitussive effect of dextromethorphan in acute URTI is marginal at best, casting doubt on its routine use in treating viral cough.

Contrary results were obtained in a recent clinical trial in children aged 6–11 years with cough associated with colds (Meeves et al., 2023) Multiple doses of dextromethorphan hydrobromide resulted in statistically significant, although modest, reductions in cough frequency and severity compared with placebo. This effect was most pronounced during the day, but there were no differences between groups in nocturnal cough frequency or sleep quality. These results suggest that dextromethorphan may have some antitussive efficacy in children, particularly for daytime cough, although its effect remains limited. Furthermore, the study highlights the importance of using objective, validated cough assessment tools in the pediatric population to increase the sensitivity of analyses of antitussive efficacy.

Other observations were reported in a study evaluating the efficacy of codeine and levodropropizine in patients with chronic cough (Lee et al., 2022). Codeine was significantly more effective than levodropropizine in reducing cough severity, as confirmed by changes in the VAS, Cough Severity Scale (CSS), and LCQ. Adverse events such as drowsiness, constipation, and headache were more frequently observed in the codeine-treated group, although these did not lead to a higher number of treatment discontinuations. These results indicate that codeine may be more effective in relieving chronic cough, but its use is associated with the risk of side effects typical of opioid medications. In contrast, levodropropizine, despite being slightly less effective, has a more favorable safety profile, making it a potentially better choice for long-term therapy.

The present review also identified several studies evaluating natural antitussive agents that could not be included in the quantitative synthesis due to substantial heterogeneity in study design, methodology, outcome measures, endpoints, and study populations.

Similarly, studies assessing natural or plant-based preparations demonstrated encouraging yet inconsistent results. Barth et al. showed that a combination of aqueous ethanolic extracts of *Justicia adhatoda* L. leaf, *Echinacea purpurea* (L.) Moench root and Eleutherococcus senticosus Harms root have an antitussive effect in patients aged 18–65 years with non-complicated URTI (Barth et al., 2015). What is more, another study (Canciani et al., 2014) confirmed that the use of Grintuss® syrup containing resins, polysaccharides, saponins, flavonoids and sugars derived from *Grindelia robusta, Plantago lanceolata, Helichrysum italicum* and honey leads to an improvement in cough symptoms in children, especially nocturnal cough, after just 4 days of use. Carnevall et al. also demonstrated that KalobaTUSS® syrup (acacia honey in combination with *Malva sylvestris, Inula helenium, Plantago major, and Helichrysum stoechas* extracts) reduced the severity and duration of daytime and nighttime cough in children with acute cough. However, it should be noted that these two studies focused on entirely different ingredients, so there is considerable risk in interpreting the results comprehensively (Carnevali et al., 2021). Another new substance was investigated in a study conducted in Iran (Ghaemi et al., 2020), which showed that lozenges containing licorice root extract (*Glycyrrhiza glabra L*.) effectively relieved symptoms of chronic cough of unknown etiology. After 2 weeks of treatment and at a four-week follow-up, a significant reduction in cough severity was observed compared to placebo, while quality of life, as assessed by the LCQ, improved.

It is also worth mentioning a study combining conventional asthma therapy with a traditional Chinese medicine preparation (Pan et al., 2025). This study demonstrated that Suhuang Zhike capsules can be an effective and safe adjunct to therapy in patients with mild to moderate asthma whose symptoms were inadequately controlled despite inhaled corticosteroids (ICS) and long-acting beta_2_-agonists (LABA) therapy. Although no significant improvement in Asthma Control Test (ACT) scores was observed in the overall population, a subgroup analysis of patients on stable treatment prior to enrollment demonstrated significant clinical improvement and reduced cough severity.

Another study (Núñez et al., 2024) showed that Sediflù syrup, containing active plant ingredients, effectively relieved cough during both the day and the night in children with upper respiratory tract infections. Significant improvement in cough severity was observed after just 2 days of treatment, and this effect persisted and intensified in subsequent days.

It should be emphasized, however, that the studies discussed in this paper differed significantly in terms of design, population, intervention type, and endpoints, which prevented their direct comparison in a meta-analysis. Some studies included preparations with mechanical or protective effects (Grintuss®, KalobaTUSS®, Sediflù), while others involved herbal products with potential anti-inflammatory or immunomodulatory effects (licorice, Suhuang Zhike, Xiehuangjiejing). The patient populations also varied, from children with acute infectious cough to patients with chronic cough or asthma, as did the intervention duration and methods for assessing efficacy (symptom scales, LCQ, VAS). Despite favorable clinical outcomes and a good safety profile, these results should be interpreted with caution because they come from studies of varying methodological quality and often a limited number of participants.

While several trials reported modest symptomatic relief, particularly in cough frequency and sleep quality, methodological weaknesses and lack of standardized outcomes considerably limit the strength and generalizability of these findings. Together, these observations underscore the urgent need for more rigorous, harmonized clinical trials with standardized outcome measures, adequate sample sizes, and improved study designs to assess better the comparative efficacy of both pharmacological and natural antitussive therapies.

The meta-analysis revealed that among the available RTCs on antitussive medications, those on P2X3 receptor antagonists demonstrated the greatest methodological consistency, particularly in the outcome measures used. Therefore, statistical analyses could be performed primarily for this group of medications, yielding reliable, comparable results on their efficacy and safety. Other interventions, due to the heterogeneity of cough assessment methods and the limited number of studies, could not be included in the quantitative analysis. This represents a significant limitation but also highlights the methodological advances in research on P2X3 antagonists.

The subgroup analysis revealed no statistically significant differences between P2X3 antagonists, indicating a consistent treatment effect across compounds. All subgroups demonstrated a reduction in cough frequency, suggesting that the observed effect is a class-related response rather than compound-specific.

After gefapixant administration, we also observed a statistically significant lower VAS score and a higher LCQ score, with a moderate, potentially clinically relevant effect and high certainty of evidence compared to placebo. In addition, after gefapixant administration, we observed that the intervention increased the risk of AEs by 41% compared with the placebo group, with low certainty of evidence. Also, sivopixant increased the risk of AEs by approximately 76% compared to the placebo group with moderate certainty of evidence. In contrast, eliapixant and camlipixant administration resulted in reductions of AEs of 2% and 15%, respectively, with low certainty of evidence. Furthermore, we noted that the type of P2X3 receptor has a significant impact on AEs frequency.

The GRADE assessment showed varying levels of evidence certainty across outcomes. Low heterogeneity, consistent effects, and narrow confidence intervals characterized high-certainty results. Moderate and low certainty were mainly due to heterogeneity, wide intervals, or a limited number of studies. Despite these differences, the overall results consistently indicated a beneficial effect of the intervention, supporting its clinical relevance.

The obtained results are consistent with previous meta-analyses and systematic reviews (Ramadan et al., 2023; Yamamoto et al., 2024), which also indicated the high efficacy of P2X3 receptor antagonists in reducing cough frequency in patients with refractory chronic cough.

From a clinical perspective, recent findings, together with our results, support the potential role of P2X3 antagonists as a targeted therapeutic option primarily for patients with refractory or unexplained chronic cough. By directly modulating cough hypersensitivity at the level of peripheral sensory pathways, these agents offer a mechanism-based, symptom-oriented approach rather than a replacement for etiological treatment, which remains the cornerstone of chronic cough management. Based on available clinical evidence, P2X3 antagonists may be best positioned as add-on or later-line therapies for patients whose symptoms persist despite optimized standard care. This treatment strategy aligns with emerging views that modulation of sensory neural pathways represents a promising adjunct approach in the management of chronic cough.

Safety and tolerability are central to the clinical implementation of P2X3 antagonists, particularly given their intended use in long-term treatment of chronic cough. In clinical studies with gefapixant, a high incidence of taste-related adverse effects has been reported, attributed to inhibition of the heteromeric P2X2/3 receptor (Yamamoto et al., 2024; Francke et al., 2025). Consequently, the development of more selective P2X3 antagonists has been pursued to improve tolerability while maintaining efficacy (Francke et al., 2025). Morice et al. (2021) showed that taste-related adverse events following eliapixant administration were less frequent at therapeutic doses than with the less selective P2X3 antagonist gefapixant. Although generally reversible upon discontinuation of treatment, taste-related adverse effects may negatively affect treatment adherence, especially during prolonged therapy. Significantly, the development of next-generation P2X3 antagonists with increased receptor selectivity has been associated with a lower incidence and severity of taste disturbances, suggesting a more favorable tolerability profile and supporting the feasibility of chronic administration. On the other hand, Morice et al. (2021) hypothesized that filapixant would be associated with a lower incidence of taste-related adverse effects compared with the non-selective P2X3/P2X2/3 antagonist gefapixant and the less selective P2X3 antagonist eliapixant. However, the results of their study were unexpected. Although filapixant was generally well tolerated, the frequency of taste-related adverse events was higher than anticipated. Therefore, further studies are required to evaluate sustained tolerability, optimal dosing strategies that balance efficacy and adverse effects, and to identify patient subgroups most likely to benefit from therapy with minimal impact on quality of life. Such data will be essential for defining the role of P2X3 antagonists within future chronic cough management algorithms and for informing clinical decision-making in routine practice.

## 5 Limitations

This meta-analysis included only pharmacological agents classified as antitussive drugs, acting directly on the cough reflex through central or peripheral mechanisms. Medications that may indirectly reduce cough by treating underlying airway inflammation or hyperresponsiveness, such as inhaled corticosteroids, leukotriene receptor antagonists, or bronchodilators, were not included. Therefore, the results primarily reflect the efficacy of classical antitussive therapy rather than broader pharmacological strategies targeting disease-specific mechanisms. This may limit the generalizability of the findings to patients whose cough is secondary to inflammatory or allergic airway disorders, such as asthma or eosinophilic bronchitis.

The available evidence may be influenced by publication bias, as studies with positive outcomes are more likely to be published, potentially overestimating the therapeutic efficacy of novel antitussive agents. Second, substantial heterogeneity exists across studies in terms of outcome measures, including differences in cough frequency assessment, patient-reported endpoints, and study duration, which limits direct comparability and quantitative synthesis of results. Finally, while P2X3 receptor antagonists are supported by the most robust clinical data to date, the number of high-quality, randomized trials evaluating alternative mechanistic targets remains limited. This imbalance limits broader conclusions about the relative efficacy of non-P2X3 therapies and underscores the need for well-designed trials across diverse antitussive strategies.

Although the literature search included both acute and chronic cough, only studies focusing on chronic cough met the inclusion criteria for quantitative synthesis. As a result, the meta-analysis and forest plots reflect evidence exclusively related to chronic cough. The absence of eligible studies on acute cough highlights a gap in the current literature and limits the generalizability of the findings to chronic cough populations only. Consequently, conclusions regarding the efficacy of P2X3 antagonists should not be extrapolated to acute cough.

Another limitation of this work is the inclusion of only English-language publications, which may have introduced a language-related selection bias. As a result, relevant studies published in other languages, particularly those originating from regions with extensive research on traditional medicine, such as China, may not have been captured. Numerous studies have investigated traditional herbal remedies with potential antitussive effects, however, many were excluded due to language restrictions and classification difficulties. Consequently, the exclusion of non-English studies and traditional herbal formulations may limit the completeness of the evidence base and should be considered when interpreting the findings of this review.

## 6 Conslusions

Our study confirms that despite the long history of use of codeine and other antitussives, both centrally (butamirate, dextromethorphan) and peripherally (levodropropizine), evidence for their effectiveness remains limited, and the available data are scattered and inconsistent. In recent years, there has been growing interest in natural preparations as alternatives to traditional antitussives. However, their efficacy and safety remain inconsistent, highlighting the need for further, high-quality clinical trials. P2X3 receptor antagonists deserve particular attention, as they have shown potential to reduce cough frequency and severity in available studies. In addition, all P2X3 antagonists reduced cough frequency, with no significant differences between compounds. Accordingly, P2X3 represents a promising direction for the development of new therapies. In this context, the concept of “beyond codeine” takes on particular significance–it encompasses not only the search for more effective and safer synthetic drugs but also the development of therapies targeting new, peripheral molecular targets, such as P2X3 receptors.

## Data Availability

The original contributions presented in the study are included in the article/Supplementary Material, further inquiries can be directed to the corresponding author.
