# Supplementary material for: Beyond codeine – the evidence landscape of conventional, natural, and emerging antitussive therapies: a systematic review and meta-analysis
Source: Front Pharmacol. 2026 Mar 2;17:1756578. doi: 10.3389/fphar.2026.1756578 (PMC12989542; doi:10.3389/fphar.2026.1756578)
Supplement: Supplementary file 1 [file Supplementaryfile1.docx]

Supplementary Material

# Supplementary Figures





**Supplementary Figure 1.** Funnel plot for the cough count (24 h frequency) after P2X3 administration compared to placebo.

Due to the small number of studies (n < 10) in individual analyses, the funnel plot was used for illustrative purposes only.





**Supplementary Figure 2.** Funnel plot VAS evaluation after gefapixant administration.

Due to the small number of studies (n < 10) in individual analyses, the funnel plot was used for illustrative purposes only.





**Supplementary Figure 3.** Funnel plot for LCQ evaluation after gefapixant administration.

Due to the small number of studies (n < 10) in individual analyses, the funnel plot was used for illustrative purposes only.





**Supplementary Figure 4.** Funnel plot for AEs assessment.

Due to the small number of studies (n < 10) in individual analyses, the funnel plot was used for illustrative purposes only.
